# The Development of A Self-Appraisal Tool for the Level of Long-Term Care Service Integration.

**DOI:** 10.1093/geroni/igab046.3083

**Published:** 2021-12-17

**Authors:** Yu-Chien Chang, Ya-Mei Chen

**Affiliations:** 1 National Taiwan University, Taiwan, Taipei, Taiwan (Republic of China); 2 National Taiwan University, Taipei, Taipei, Taiwan (Republic of China)

## Abstract

Introduction Taiwan is the fastest aging countries in the world. In 2016, Taiwan implemented Long-Term Care Plan 2.0 (LTC Plan 2.0), aims to provide coordinated and integrated LTC services. However, how to assess the level of integration and which integration mechanisms are better applied are still unclear in the literature. This study intended to address a research question regarding “How can agencies measure their level of service integration?” and, therefore, aimed to develop an integration assessment tool—the Taiwanese Self-Assessment for LTC Systems Integration (TwSASI)- for LTC agencies to use to self-evaluate their current “level” of providing integrating LTC services. Methods 　　TwSASI was first developed base on Connie J. Evashwick’s (2005) framework and literature review, including four domains: inter-entity planning and management, care coordination, integrated information system, and integrated financing, and 11 dimensions with 51 items. Through the Delphi method, with two rounds of investigation and feedback from 26 experts, RAND/UCLA Appropriateness Method (RAM) was used to assess the consensus regarding the dimensions and items developed and refined the tool content accordingly. Results After two rounds of investigation, four domains remained with 10, 11, 4, and 5 items in each domain respectively. All items reached good experts' consensus with medians of the 30 items’ importance, feasibility, and appropriateness all over 8. The Scale Content Validity Index (SCVI) of the 4 dimensions all over than 0.9. Conclusion The TwSASI can be feasible for evaluating the level of LTC service integration in Taiwan. LTC agencies can improve their level of service integration accordingly.

